# Co-Produced Research Priorities for Metabolic Interventions in Severe Mental Illness: A James Lind Alliance Priority Setting Partnership

**DOI:** 10.1192/bjo.2026.11242

**Published:** 2026-06-30

**Authors:** Dina Farran, Toby Pillinger, Fiona Gaughran, Khalida Ismail

**Affiliations:** King’s College London, London, United Kingdom

## Abstract

**Aims:**

Metabolic dysfunction is a major driver of excess morbidity and premature mortality in people with severe mental illness (SMI). Converging evidence suggests that disturbances in brain energy metabolism may contribute to psychiatric symptoms in SMI. Despite growing interest in metabolic psychiatry, research in this area has largely been shaped by academic priorities, with limited structured input from people with lived experience, carers, or front-line professionals regarding which metabolic interventions and outcomes matter most to them. Consequently, evidence to guide metabolic approaches that improve physical and mental health outcomes in SMI remains fragmented and poorly aligned with stakeholder priorities. The James Lind Alliance (JLA) Priority Setting Partnership (PSP) for Metabolic Interventions in SMI was established to identify and prioritise research uncertainties that matter most to people with lived experience, carers, and professionals.

**Methods:**

The PSP followed the five-stage JLA methodology: defining scope, gathering questions, refining and verifying uncertainties, interim prioritisation, and a final consensus workshop. An international open-call survey collected research questions from people with lived experience of SMI, carers, and professionals. Submissions were thematically synthesised into summary questions and checked against evidence through an umbrella review of meta-analyses to identify unanswered uncertainties. Verified uncertainties were ranked in an online survey. A structured, facilitated online workshop using nominal group techniques produced the final Top 10 priorities.

**Results:**

In the first survey, 456 participants submitted 1,439 research questions. After removal of out-of-scope submissions and qualitative synthesis, 51 summary questions across 13 thematic domains were generated. Evidence checking identified two questions as adequately answered, leaving 49 verified uncertainties. In the interim prioritisation survey, 317 participants ranked these uncertainties, producing a shortlist of 19 questions. Thirty stakeholders (16 with lived experience or carers; 14 professionals) took part in the final online workshop. The resulting Top 10 priorities focused on dietary interventions, combined lifestyle strategies, GLP-1 receptor agonists for medication-related weight gain and potential mental health benefits, micronutrient deficiencies, physical activity, metabolic effects of psychiatric medications beyond weight gain, gut-brain mechanisms, brain changes associated with metabolic improvement, and the role of ketone bodies in symptom improvement and remission.

**Conclusion:**

This PSP establishes the first stakeholder-driven research agenda for metabolic psychiatry in SMI, identifying priorities that integrate diet, physical activity, pharmacological adjuncts, and mechanistic pathways linking metabolic and mental health. The Top 10 priorities provide a translational roadmap for future trials, mechanistic studies, and implementation research aimed at reducing cardiometabolic inequalities and improving psychiatric outcomes in SMI.

This work was funded by UK Research and Innovation (UKRI) through the Hub for Metabolic Psychiatry.

